# The dynamics of RUNX1-RUNX1T1 transcript levels after allogeneic hematopoietic stem cell transplantation predict relapse in patients with t(8;21) acute myeloid leukemia

**DOI:** 10.1186/s13045-017-0414-2

**Published:** 2017-02-06

**Authors:** Ya-Zhen Qin, Yu Wang, Lan-Ping Xu, Xiao-Hui Zhang, Huan Chen, Wei Han, Yu-Hong Chen, Feng-Rong Wang, Jing-Zhi Wang, Yao Chen, Xiao-Dong Mo, Xiao-Su Zhao, Ying-Jun Chang, Kai-Yan Liu, Xiao-Jun Huang

**Affiliations:** 1 Peking University People’s Hospital, Peking University Institute of Hematology, Beijing Key Laboratory of Hematopoietic Stem Cell Transplantation, Beijing, 100044 China; 2 Peking-Tsinghua Center for Life Sciences, Beijing, 100871 China

**Keywords:** RUNX1-RUNX1T1 transcript levels, Acute myeloid leukemia, Allogeneic hematopoietic stem cell transplantation, Relapse, Donor lymphocyte infusion

## Abstract

**Background:**

The optimal monitoring schedules and cutoff minimal residual disease (MRD) levels for the accurate prediction of relapse at all time points after allogeneic hematopoietic stem cell transplantation (allo-HSCT) remain unclear in patients with t(8;21) acute myeloid leukemia (AML).

**Methods:**

RUNX1-RUNX1T1 transcript levels were measured in bone marrow samples collected from 208 patients at scheduled time points after transplantation (1530 samples in total).

**Results:**

A total of 92.3% of the requested samples were collected, and 74.0% of patients had complete sample collection. The 1-, 3-, and 6-month RUNX1-RUNX1T1 transcript levels could significantly discriminate between continuous complete remission and a hematologic relapse at 1.5–3, 4–6, and 7–12 months but not at >3, >6, and >12 months, respectively. Over 90% of the 175 patients who were in continuous complete remission had a ≥3-log reduction in RUNX1-RUNX1T1 transcript levels from the time of diagnosis at each time point after transplantation and a ≥4-log reduction at ≥12 months. A <3-log reduction within 12 months and/or a <4-log reduction at ≥12 months was significantly related to a higher 3-year cumulative incidence of relapse (CIR) rate in both the entire cohort and the patients with no intervention after HSCT (58.4 vs. 2.2%, 76.5 vs. 2.0%; all *P* < 0.0001). Patients who had received a preemptive donor lymphocyte infusion when the increase in RUNX1-RUNX1T1 transcripts was ≤1-log according to the above dual cutoff values had significantly lower 1-year CIR rate after intervention than the patients who had received an infusion when the increase was >1-log (0 vs. 55.0%, *P* = 0.015).

**Conclusions:**

RUNX1-RUNX1T1 transcripts with a <3-log reduction from diagnosis within 12 months and/or a <4-log reduction at ≥12 months after allo-HSCT could accurately predict relapse and may prompt a timely intervention in patients with t(8;21) AML.

**Electronic supplementary material:**

The online version of this article (doi:10.1186/s13045-017-0414-2) contains supplementary material, which is available to authorized users.

## Background

Although t(8;21) acute myeloid leukemia (AML) is considered to have a good prognosis, relapse occurs in up to 40% of patients treated with chemotherapy [1–6]. Allogeneic hematopoietic stem cell transplantation (allo-HSCT) is a potentially curative approach for patients with acute myeloid leukemia (AML) [7–9]. High-risk t(8;21) AML patients benefited from allo-HSCT in our previously published report [6]. Even after they had received HSCT, 10–20% of patients still experienced relapse, which led to a poor long-term outcome [10, 11].

In patients with t(8;21) AML, the presence of minimal residual disease (MRD), measured by RUNX1-RUNX1T1 transcript levels, is now established as a powerful marker to predict relapse and to direct clinical interventions for patients receiving chemotherapy or transplantation [4–6, 10, 12–16]. Our previous study indicated that a <3-log reduction from diagnosis in RUNX1-RUNX1T1 transcripts at the first 3 months post-HSCT generally identified high relapse risk patients [12].

Additionally, we realized that patients with an early ≥3-log reduction in RUNX1-RUNX1T1 transcripts might experience late relapse post-HSCT. Regarding the prediction of relapse that occurs at all time points after HSCT, only a few case reports and studies with a very small sample size have shown that an increase in the number of RUNX1-RUNX1T1 transcripts occurred prior to hematologic relapse [16–18]. Due to the low incidence of t(8;21) AML [19] and its low proportion of patients undergoing allogeneic transplantation, no large-scale study results have been presented to date. As a result, the exact threshold of MRD levels and monitoring schedules for the accurate prediction of relapse at all time points after allo-HSCT remain unclear.

In the current study, we evaluated the RUNX1-RUNX1T1 transcript levels in 208 patients who were monitored at scheduled time points after HSCT and found that a <3-log reduction from diagnosis within 12 months and/or a <4-log reduction at ≥12 months accurately predicted relapse at all time points after HSCT and led to an effective preemptive donor lymphocyte infusion (DLI).

## Methods

### Patients, treatment, and samples

A total of 208 t(8;21) AML patients were included in this study. They were in complete remission (CR, first or second) at the time of HSCT and consecutively received allo-HSCT at our institute from March 2006 to May 2016. All patients received induction therapy (one to four courses) to achieve CR, followed by at least 2 cycles of consolidation therapy before receiving a transplant. Indications for allo-HSCT included (1) hematologic relapse, (2) meeting the high-risk criteria that we published (not achieving a ≥3-log reduction after the second consolidation and/or the loss of a ≥3-log reduction during the next six consolidation therapies) [6], (3) c-KIT mutations at the time of diagnosis [20], and (4) the patient’s repeated request. The allo-HSCT conditioning regimen, graft-versus-host disease (GVHD) prophylaxis, modified DLI regimen, and preemptive interferon-a (IFN-a) treatment for MRD were performed as previously described [8, 21, 22]. All patients who received haploidentical, unrelated or cord blood grafts were administered an oral dose of rabbit anti-thymocyte globulin (ATG, 2.5 mg/kg; Sanofi, Gentilly, France) on days five to two before transplantation. A modified DLI or IFN-a were given before hematological relapse as intervention therapy after 3 months post-HSCT following a trial of immunosuppressant withdrawal if patients did not achieve a ≥3-log reduction of RUNX1-RUNX1T1 until 3 months or lost a ≥3-log reduction after HSCT without uncontrolled GVHD or severe infection according to donor availability and willingness.

The monitoring schedule was planned in advance, and the scheduled time points were 0, 1, 2, 3, 4.5, 6, 9, 12, 18, and 24 months post-HSCT. Morphologic evaluations and the quantitative measurement of RUNX1-RUNX1T1 transcripts in bone marrow (BM) samples were performed at the specified time points and when patients showed signs of relapse. The cutoff date for follow-up was August 15, 2016. The median follow-up time after HSCT was 24 (2–89) months in the entire cohort. The study was approved by the Ethics Committee of Peking University People’s Hospital, and all patients or their guardians provided written informed consent to participate in the study in accordance with the Declaration of Helsinki.

### Measurement of RUNX1-RUNX1T1 transcript levels

TRIzol reagent (Invitrogen, CA, USA) was used to extract total RNA. A high capacity cDNA reverse transcription kit (Applied Biosystems, Foster City, CA, USA) was used to synthesize complementary DNA (cDNA). TaqMan-based real-time quantitative PCR (RQ-PCR) technology was used as described previously [6, 7, 17]. The primers and probes for ABL and RUNX1-RUNX1T1 were obtained from the report of the Europe Against Cancer Program [23, 24]. Quality control samples were included in each PCR run. All amplifications were performed at least in duplicate. The RUNX1-RUNX1T1 transcript level was calculated as the percentage of RUNX1-RUNX1T1 transcript copies/ABL copies. The pretreatment baseline level of RUNX1-RUNX1T1 transcripts was 400% in our laboratory [6]. The reproducible sensitivity of RQ-PCR is five copies. All of the samples with an undetectable fusion transcript had ≥12,500 copies of ABL to guarantee that at least a 4-log reduction of RUNX1-RUNX1T1 transcript levels (0.04%) could be detected.

### Statistical analysis and definitions

The cumulative incidence of relapse (CIR), disease free survival (DFS), and overall survival (OS) were measured from the time of allo-HSCT. Standard definitions of relapse were used. Relapses included BM and/or extra-medullary sites. The events for measuring DFS included death and relapse. The event for measuring OS was death (regardless of the cause), and patients, or their relatives, were queried at the date of last follow-up to determine whether they were still living or censored on the date that they were last known to be alive. Comparisons of the CIR between the groups were calculated by considering the competing risks defined by death and performed with the Gray test. The molecular response (the log reduction of RUNX1-RUNX1T1 transcript levels) was considered a time-dependent covariate, i.e., the start time point of the CIR rate curves according to the transcript levels at a specific time point was that specific time point. Survival functions were estimated using the Kaplan-Meier method and compared using the log-rank test. Comparisons between the two groups were performed using the Mann-Whitney *U* test for continuous variables and Fisher’s exact test for categorical variables. Receiver operating characteristic (ROC) curves were used to evaluate the effect of RUNX1-RUNX1T1 transcript levels after HSCT on relapse. The level for a statistical significance was set at *P* ≤ 0.05. R version 2.6.1 (R Foundation for Statistical Computing, Vienna, Austria), SPSS 13.0 (SPSS Inc., Chicago, IL, USA), and GraphPad Prism 5 (GraphPad Software Inc., La Jolla, CA, USA) software were used.

## Results

### Patient characteristics, outcomes, and time of relapse

Patient characteristics at the time of diagnosis and HSCT are shown in Table 1. After receiving allo-HSCT, 33 (15.9%) patients experienced relapse; 25 and 8 patients suffered only hematologic and extra-medullary relapse, respectively. A total of 156 (75.0%) patients were alive at the last follow-up; a total of 22 patients died of relapse, and 30 died due to treatment-related mortality. The median follow-up time after allo-HSCT was 26 (3–89) months for the surviving patients. The 3-year CIR, DFS, and OS rates were 20.4% (95% confidence interval (CI), 9.9–33.6%), 65.4% (95% CI, 57.4–72.2%), and 70.8% (95% CI, 62.9–77.3%), respectively.

**Table 1:** Patient characteristics

Parameters	*n* = 208
Median age when receiving HSCT, y (range)	30 (4–57)
Gender	
Male	124 (60%)
Female	84 (40%)
c-KIT gene at diagnosis	
Mutation	69 (33%)
Wild type	80 (39%)
Unknown	59 (28%)
First CR induction courses	
1	136 (65%)
>1	58 (28%)
Unknown	14 (7%)
Interval between diagnosis and HSCT	
<8 months	110 (53%)
≥8 months	98 (47%)
Disease status when receiving HSCT	
First CR	179 (86%)
Second CR	29 (14%)
Donor source	
HLA-matched sibling	60 (29%)
Haploidentical	135 (65%)
Unrelated donor	10 (5.5%)
Cord blood	1 (0.5%)
Conditioning regimen	
Chemotherapy based	204 (98.1%)
TBI based	4 (1.9%)

The median relapse time point was 6 months (range 1.5–43 months) after HSCT for 33 relapsed patients. A total of 27 (82%) patients experienced relapse within the first year after HSCT (11 at 1.5–3 months, 6 at 4–6 months, and 10 at 7–12 months), and the remaining 6 (18%) patients relapsed after 12 months (at 26–43 months after HSCT).

### Sample collection and monitoring implementation

Seventy-four percent (154/208) of patients were collected of all requested samples with qualified ABL copies. Of the 54 patients with incomplete collections, 30 (55.6%) and 14 (25.9%) lacked 1 and 2 qualified requested samples, respectively. In all, RUNX1-RUNX1T1 transcripts were measured in 1530 BM samples in this study, which accounted for 92.3% of the requested number of samples.

### A <3-log reduction from diagnosis in RUNX1-RUNX1T1 transcripts within the first 3 months after HSCT predicted relapse

**Fig. 1:**
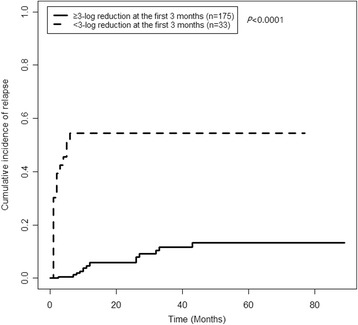
CIR rates of the patients categorized according to their RUNX1-RUNX1T1 transcript levels at the first 3 months after HSCT

Patients with a <3-log reduction in RUNX1-RUNX1T1 transcripts at 1–3 months after HSCT had a significantly higher 3-year CIR rate than those with a ≥3-log reduction (54.5% [95% CI, 40.3–70.8%]) vs. 11.6% [95% CI, 3.0–31.5%]; *P* < 0.0001, Fig. 1).

### A <3-log reduction in RUNX1-RUNX1T1 transcripts within the first 3 months was relevant to early but not to late relapse

**Fig. 2:**
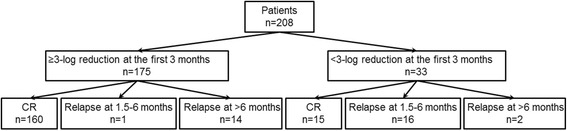
Distribution of the patients according to their reduction in RUNX1-RUNX1T1 transcript levels at 1–3 months and relapse time after allo-HSCT

Figure 1 shows that some of the patients who had achieved a ≥3-log reduction in RUNX1-RUNX1T1 transcripts at 1–3 months still suffered a relapse. The effect of the early MRD level on relapse was further analyzed in terms of relapse time. The distributions of patients are shown in Fig. 2. A <3-log reduction in RUNX1-RUNX1T1 transcripts at 1–3 months was significantly associated with a higher rate of relapse at 1.5–6 months than a ≥3-log reduction (relapses after 6 months were excluded. 16/31 vs. 1/161, 51.6 vs. 0.6%, *P* < 0.0001); a <3-log reduction in RUNX1-RUNX1T1 transcripts was not related to relapse after 6 months (relapses at 1.5–6 months were excluded. 2/17 vs. 14/174, 11.8 vs. 8.0%, *P* = 0.64).

### The RUNX1-RUNX1T1 transcript levels after HSCT only predicted the forthcoming relapse

ROC curve analysis was performed. To evaluate the effect of the 1-month RUNX1-RUNX1T1 transcript levels on relapse, the patients were categorized into three groups: continuous CR, relapse at 1.5–3 months, and relapse after 3 months. The analysis demonstrated that the 1-month RUNX1-RUNX1T1 transcript levels could significantly discriminate continuous complete remission (CR) from relapse at 1.5–3 months (area under the curve [AUC] 0.85, *P* < 0.0001), but not relapse after 3 months (AUC 0.53, *P* = 0.62). Similarly, the 3-month RUNX1-RUNX1T1 levels significantly discriminated continuous CR from relapse at 4–6 months (AUC 0.98, *P* < 0.0001), but not at >6 months (AUC 0.60, *P* = 0.24). In addition, the 6-month RUNX1-RUNX1T1 levels could discriminate continuous CR from relapse at 7–12 months (AUC 0.75, *P* = 0.009), but not after 12 months (AUC 0.50, *P* = 0.98).

**Fig. 3:**
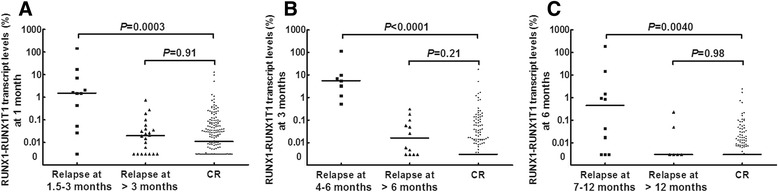
Comparisons of the RUNX1-RUNX1T1 transcript levels at 1, 3, and 6 months among the patients in continuous CR, the patients who had experienced forthcoming relapse and the patients who had experienced late relapse. **a** RUNX1-RUNX1T1 transcript levels at 1-month post-HSCT. **b** RUNX1-RUNX1T1 transcript levels at 3 months post-HSCT. **c** RUNX1-RUNX1T1 transcript levels at 6 months post-HSCT

The RUNX1-RUNX1T1 transcript levels at 1, 3, and 6 months were individually compared among the patients in continuous CR, the patients who had experienced forthcoming relapse, and the patients who had experienced late relapse. At 1 month, the RUNX1-RUNX1T1 transcript levels in the patients in continuous CR were significantly lower than in the patients who had relapsed at 1.5–3 months, but similar levels to that in the patients who had relapsed after 3 months (Fig. 3a). Likewise, at 3 months, the CR patients had significantly lower RUNX1-RUNX1T1 transcript levels than those who had relapsed at 4–6 months, but similar levels to those patients who had relapsed after 6 months (Fig. 3b). At 6 months, the CR patients had significantly lower RUNX1-RUNX1T1 transcript levels than those who had relapsed at 7–12 months but had levels that were similar to those patients who had relapsed after 12 months (Fig. 3c).

These results showed that RUNX1-RUNX1T1 transcript levels after HSCT only predicted forthcoming relapse but not late relapse. Therefore, serial monitoring of RUNX1-RUNX1T1 transcripts after HSCT is needed for the accurate prediction of relapse at any time.

### Dynamic patterns of RUNX1-RUNX1T1 transcript levels after HSCT in patients in continuous CR

As shown in Table 2, for the patients in continuous CR (*n* = 175), the RUNX1-RUNX1T1 transcript levels gradually decreased after HSCT, which was demonstrated by the gradual reduction of the median RUNX1-RUNX1T1 transcript levels and the increase of the percentage of patients with a ≥3-log and a ≥4-log reduction in RUNX1-RUNX1T1 transcripts. Over 90% of patients had a ≥3-log reduction from the first month and had a ≥4-log reduction at ≥12 months post-HSCT. In addition, these two frequencies were similar at each time point starting at 12 months (*P* > 0.05). Therefore, ≥3-log and ≥4-log reductions in RUNX1-RUNX1T1 transcripts were individually chosen as the thresholds for the prediction of relapse within the first year and starting at 12 months post-HSCT, respectively.

**Table 2:** Dynamics of RUNX1-RUNX1T1 transcripts post-HSCT in patients in continuous CR

Month post-HSCT	Number of patients evaluated	Median RUNX1-RUNX1T1 transcript levels (range)	Patients with ≥3-log reduction (%)	Patients with ≥4-log reduction (%)	*P* value*
1	174	0.011% (0–12.8%)	163 (93%)	121 (70%)	<0.0001
2	167	0.0062% (0–22.5%)	160 (96%)	132 (79%)	<0.0001
3	161	0 (0–17.8%)	153 (95%)	127 (79%)	<0.0001
4.5	126	0 (0–1.5%)	122 (97%)	93 (74%)	<0.0001
6	143	0 (0–2.4%)	139 (97%)	123 (86%)	0.001
9	113	0 (0–4.1%)	111 (98%)	99 (88%)	0.003
12	118	0 (0–0.66%)	116 (98%)	110 (93%)	0.10
18	90	0 (0–0.30%)	90 (100%)	87 (97%)	0.25
24	72	0 (0–0.30%)	72 (100%)	68 (94%)	0.12
30	23	0 (0–0.49%)	22 (96%)	22 (96%)	1.0
36	27	0 (0–0.005%)	27 (100%)	27 (100%)	1.0

*Comparison of the frequency of patients with a ≥3-log reduction with the frequency of patients with a ≥4-log reduction at each time point

### A <3-log reduction within 12 months and/or a <4-log reduction at ≥12 months after HSCT predicted relapse at all time points after allo-HSCT

**Fig. 4:**
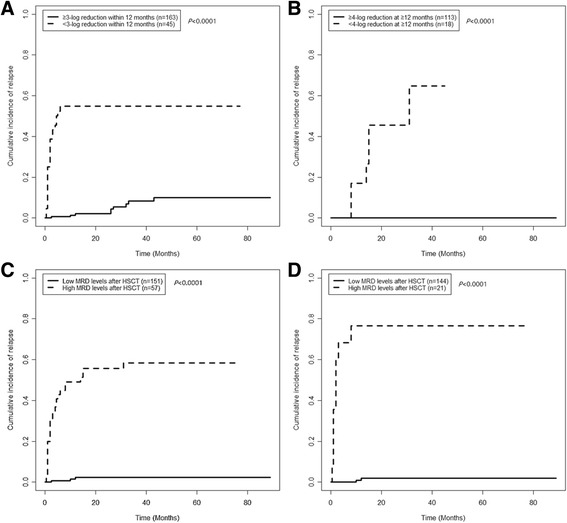
Comparisons of the CIR rates among patients with different MRD levels after HSCT. **a** The patients were grouped according to whether they achieved a ≥3-log reduction within 12 months. **b** The patients were grouped according to whether they achieved a ≥4-log reduction at ≥12 months. **c** All of the patients were grouped according to the dual cutoff values. **d** The patients with no intervention after HSCT were grouped according to the dual cutoff values

As shown in Fig. 4, both a <3-log reduction in RUNX1-RUNX1T1 transcripts within 12 months and a <4-log reduction in RUNX1-RUNX1T1 transcripts at ≥12 months post-HSCT significantly predicted relapse, respectively (Fig. 4a and b, all *P* < 0.0001). In addition, patients who had a ≥3-log reduction in RUNX1-RUNX1T1 transcripts within 12 months and a ≥4-log reduction at ≥12 months (defined as low MRD levels after HSCT, *n* = 151) have significantly lower 3-year CIR rates than patients with a <3-log reduction within 12 months and/or a <4-log reduction at ≥12 months (defined as high MRD levels after HSCT, *n* = 57) in the entire cohort (2.2% [95% CI, 0–30.6%] vs. 58.4% [95% CI, 45.5–68.7%]; *P* < 0.0001; Fig. 4c). This result was also observed in the patients with no intervention after HSCT (*n* = 144 and 21, 2.0% [95% CI, 0–36.5%] vs. 76.5% [95% CI, 74.9–85.5%]; *P* < 0.0001; Fig. 4d).

### A ≤1-log increase according to the dual thresholds directed effective preemptive DLI

**Fig. 5:**
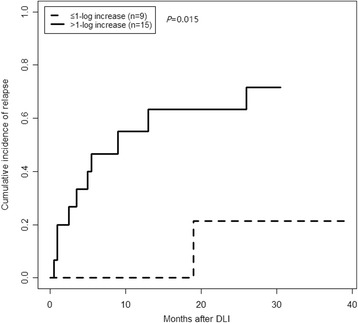
CIR rates of the patients grouped by the increase in RUNX1-RUNX1T1 transcripts compared with the dual thresholds at the time of preemptive DLI

Of the 43 patients who were given intervention therapy, 24 received preemptive DLI (12 also received IFN-a) for the prevention of hematologic relapse. The median time point at which DLI was received was 6 months (range 3–26 months) post-HSCT, and the median RUNX1-RUNX1T1 transcript level was 3.1% (range 0.12–163.0%) at the time of the intervention. The patients were categorized according to their RUNX1-RUNX1T1 transcript levels at the time of DLI. Nine patients received preemptive DLI when their RUNX1-RUNX1T1 transcripts were increased by ≤1-log according to the above dual thresholds (that is, a 2–3-log reduction from diagnosis in RUNX1-RUNX1T1 transcripts within 12 months [*n* = 8] or a 3–4-log reduction at ≥12 months [*n* = 1]), and 15 patients received preemptive DLI when their increase was >1-log (<2-log reduction within 12 months [*n* = 11] or <3-log reduction at ≥12 months [*n* = 4]). Figure 5 reveals that the patients in the former group had significantly lower 1-year CIR rate after intervention than the patients in the latter group (0 vs. 55.0% [95% CI, 33.9–68.7%]; *P* = 0.015). This finding indicates that if the cutoff values for prompting preemptive DLI were increased by 1-log, it could no longer effectively prevent relapse.

### The impact of factors other than the RUNX1-RUNX1T1 transcript levels after HSCT on relapse

As shown in Table 3, mutated c-KIT gene, HLA-matched sibling donor and <3-log reduction of RUNX1-RUNX1T1 transcript levels pre-HSCT were significantly related to a higher 3-year CIR rate (all *P* < 0.05), and second CR status pre-HSCT tended to be significantly related to a higher 3-year CIR rate (*P* = 0.071).

**Table 3:** The impact of factors other than the RUNX1-RUNX1T1 transcript levels after HSCT on relapse

Factors	3-year CIR rate (95% CI)	*P* value
WBC count at diagnosis		
≤ 10 × 10^9^/L	24.6% (9.1–44.0%)	0.31
> 10 × 10^9^/L	17.1% (5.1–35.0%)	
c-KIT gene		
Mutation	38.4% (20.0–56.6%)	*0.003*
Wild type	15.4% (2.4–38.9%)	
Karyotype		
Sole t(8;21)	20.6% (4.3–45.4%)	0.56
Additional abnormalities	21.6% (7.6–40.3%)	
Course acquired to achieve CR		
1	19.0% (6.1–372%)	0.20
> 1	24.2% (7.9–45.3%)	
Time interval from diagnosis to transplant		
< 8 months	23.5% (9.4–41.2%)	0.87
≥ 8 months	16.4% (4.3–35.5%)	
Disease status pre-HSCT		
1st CR	18.8% (7.7–33.6%)	0.071
2nd CR	33.3% (9.0–60.4%)	
Donor resource		
HLA-matched sibling	31.4% (13.5–51.2%)	*0.039*
Alternative donor	14.6% (4.2–31.0%)	
RUNX1-RUNX1T1 transcript levels pre-HSCT		
≥ 3-log reduction	11.1% (0.9–36.4%)	*<0.0001*
< 3-log reduction	27.9% (14.6–42.9%)	
Acute GVHD		
With	21.6% (7.5–40.5%)	0.52
Without	18.9% (6.0–37.3%)	

Statistically significant factors are italicized

The impact of acute GVHD on the evolution of the RUNX1-RUNX1T1 transcript levels is shown in the Additional file 1.

## Discussion

MRD-directed therapy is a new treatment strategy for patients with AML who receive chemotherapy as well as transplantation. Determination of the cutoff MRD value and the monitoring schedule is the present challenge. In the current study, we demonstrated that the dynamics of RUNX1-RUNX1T1 transcript levels accurately predicted relapse after allo-HSCT in patients with t(8;21) AML. Furthermore, MRD monitoring results directed the preemptive use of DLI, which effectively decreased the occurrence of a hematological relapse.

t(8;21) is a rare disease that accounts for approximately 8% of AML cases [17]. In addition, because t(8;21) is defined as a favorable characteristic, only high-risk patients have been recommended to receive allo-HSCT in recent years [6]. As a result, all of the studies concerning MRD in t(8;21) AML patients who received an allo-HSCT had small sample sizes or were case reports [15–17], except for the study that we published in 2014 [10]. In that paper, we described the effect of changes in RUNX1-RUNX1T1 transcript levels in the first 3 months after allo-HSCT on relapse prediction. To date, no studies have investigated the optimal MRD thresholds and the monitoring schedules for the prediction of relapse at all time points after allo-HSCT in patients with t(8;21) AML.

Several studies have individually shown that a 3-log reduction in RUNX1-RUNX1T1 transcripts is a meaningful threshold at distinct time points during chemotherapy [4–6]. Regarding transplantation, our multicenter data demonstrated that a ≤3-log reduction at the first 3 months after HSCT was an independent factor for CIR, which could be used to rapidly identify those at high risk of relapse [10].

In the present study, we confirmed the predictive value of a 3-log reduction in RUNX1-RUNX1T1 transcripts in the first 3 months for relapse in the entire cohort. However, despite the statistical significance, the majority of late relapse events could not be predicted by the MRD status within the first 3 months. Both the ROC curve analysis and the comparisons of the RUNX1-RUNX1T1 transcripts showed that 1-, 3, and 6-month RUNX1-RUNX1T1 transcript levels could significantly discriminate continuous CR from hematologic relapse that occurred at 1.5–3, 4–6, and 7–12 months but not at >3, >6, or >12 months, respectively. This finding suggested that RUNX1-RUNX1T1 transcript levels after HSCT only predict forthcoming relapse. Therefore, the serial monitoring of RUNX1-RUNX1T1 transcript levels is needed to predict the occurrence of relapse at all time points after HSCT.

Previous studies have demonstrated that a rapid increase in RUNX1-RUNX1T1 transcripts was typically found before relapse occurred [13–18]. This finding suggested that the dynamics of RUNX1-RUNX1T1 after HSCT might predict a relapse. However, the cutoff values and the monitoring time points needed to be determined. In the current study, these determinations were made by the analysis of RUNX1-RUNX1T1 dynamics in patients who were maintained in continuous CR after HSCT. We found that over 90% of patients exhibited a ≥3-log reduction and a ≥4-log reduction in RUNX1-RUNX1T1 transcripts from the first month and at ≥12 months, respectively. Furthermore, the presence of similar frequencies at ≥12 months reflected that these two cutoff values categorized similar amounts of patients into the continuous CR group. Because the cutoff value selection criteria were designed to categorize the highest number of poor-outcome patients into the high-risk group, we developed dual cutoff values: a 3-log reduction for the first year and a 4-log reduction thereafter. As expected, these values accurately predicted relapse in the entire cohort as well as in patients who had received no intervention.

Our previous paper demonstrated that interventions with preemptive DLI decreased the rate of post-HSCT relapse in patients with t(8;21) AML [10]. However, the threshold of RUNX1-RUNX1T1 transcripts for the implementation of an effective intervention remains unclear. In this study, we found that the CIR rate of patients who received DLI when their RUNX1-RUNX1T1 transcripts were within a 1-log increase from the dual thresholds was significantly lower than that of patients receiving DLI at the time of a >1-log increase. Therefore, the effectiveness of preemptive DLI is relevant to RUNX1-RUNX1T1 transcript level at the time of the intervention. A DLI could not prevent hematologic relapse once the leukemic burden was too high. The results demonstrated that the dual cutoff values that we established may direct a timely and effective intervention. However, this result must be confirmed in more cases.

Apart from the RUNX1-RUNX1T1 transcript levels after HSCT, c-KIT mutation status, donor type, and RUNX1-RUNX1T1 transcript levels pre-HSCT were also found to be related to relapse after HSCT in the current cohort. Previous studies have shown the prognostic value of these factors in AML. The c-KIT mutation is a strong poor prognostic factor in t(8;21) AML [6, 10, 20, 25]. MRD levels before allogeneic HSCT have been demonstrated to be associated with adverse outcomes in AML by many studies [26, 27]. We previously reported that high-risk acute leukemia patients receiving haploidentical donor grafts had a significantly lower relapse rate than those receiving HLA-identical sibling donor grafts [28].

The monitoring schedule of this study was planned in advance. In patients who had received allo-HSCT, relapse mostly occurred within the first year, and the relapse rate was highest within the first 3 months; the time interval was therefore set at 1 month within the first 3 months and gradually increased thereafter. A recent report on prospective monitoring in t(8;21) AML patients showed that 71.3% of BM samples that were planned by the protocol were collected [14]. In the present study, 92.3% of the planed samples were collected, and 74.0% of the patients followed the schedule. This finding implied that the results of the current study could represent the effect of this monitoring schedule, and such scheduled monitoring effectively predicted relapse.

## Conclusions

The results of the present study indicate that monitoring the dynamics of RUNX1-RUNX1T1 transcripts could predict relapse at all time points after allo-HSCT in patients with t(8;21) AML. A <3-log reduction and a <4-log reduction from diagnosis in RUNX1-RUNX1T1 transcripts within 12 months and at ≥12 months post-HSCT, respectively, were the optimal thresholds to predict relapse. A <1-log increase relative to the above dual thresholds directed the timely and effective use of preemptive DLI. These results should be confirmed in a multi-center study.

## Additional file

Additional file 1: Impact of acute GVHD on the evolution of RUNX1-RUNX1T1 transcript levels. (DOCX 15 kb)

## Abbreviations

allo-HSCT: Allogeneic hematopoietic stem cell transplantation
AML: Acute myeloid leukemia
AUC: Area under the curve
CI: Confidence interval
CIR: Cumulative incidence of relapse
DFS: Disease-free survival
DLI: Donor lymphocyte infusion
GVHD: Graft-versus-host disease
IFN-a: Interferon-a
MRD: Minimal residual disease
OS: Overall survival
RQ-PCR: Real-time quantitative PCR
