# Supplementary material for: The dynamics of RUNX1-RUNX1T1 transcript levels after allogeneic hematopoietic stem cell transplantation predict relapse in patients with t(8;21) acute myeloid leukemia
Source: J Hematol Oncol. 2017 Feb 6;10:44. doi: 10.1186/s13045-017-0414-2 (PMC5294828; doi:10.1186/s13045-017-0414-2)
Supplement: Additional file 1: — Impact of acute GVHD on the evolution of RUNX1-RUNX1T1 transcript levels. (DOCX 15 kb) [file 13045_2017_414_MOESM1_ESM.docx]

**Supplement**

**Impact of acute GVHD on the evolution of RUNX1-RUNX1T1 transcript levels**

A total of 60 patients could not be analyzed because their RUNX1-RUNX1T1 transcript levels were 0% at all time points before and after acute GVHD or because no samples were collected after GVHD. Of the remaining 148 evaluable patients, 68 had Grade I-IV acute GVHD, and their RUNX1-RUNX1T1 transcript levels decreased in 40 (59%) and increased in 28 (41%) after GVHD. In addition, 80 patients had no acute GVHD and served as a control group, and their RUNX1-RUNX1T1 transcript levels decreased in 38 (48%) and increased in 42 (52%) during the first 3 months after HSCT. As a result, acute GVHD was significantly related to the decrease of RUNX1-RUNX1T1 transcript levels in the current cohort (*P*=0.019). Because of the incomplete data, we could not perform a thorough analysis of the impact of chronic GVHD on the evolution of RUNX1-RUNX1T1 transcript levels.
